# Impact of Frequent Administration of Bacteriophage on Therapeutic Efficacy in an *A. baumannii* Mouse Wound Infection Model

**DOI:** 10.3389/fmicb.2020.00414

**Published:** 2020-03-17

**Authors:** Michael D. Rouse, Joshua Stanbro, Jessica A. Roman, Michelle A. Lipinski, Anna Jacobs, Biswaijt Biswas, James Regeimbal, Matthew Henry, Michael G. Stockelman, Mark P. Simons

**Affiliations:** ^1^Henry M Jackson Foundation for the Advancement of Military Medicine, Bethesda, MD, United States; ^2^Naval Medical Research Center, Silver Spring, MD, United States; ^3^Walter Reed Army Institute of Research, Silver Spring, MD, United States; ^4^Biological Defense Research Directorate, Naval Medical Research Center, Fort Detrick, MD, United States; ^5^Naval Medical Research Unit-6, Lima, Peru

**Keywords:** bacteriophage, *Acinetobacter baumannii*, wound infection, host immunity, therapeutic efficacy

## Abstract

The spread of multidrug antibiotic resistance (MDR) is a widely recognized crisis in the treatment of bacterial infections, including those occurring in military communities. Recently, the World Health Organization published its first ever list of antibiotic-resistant “priority pathogens” – a catalog of 12 families of bacteria that pose the greatest threat to human health with *A. baumannii* listed in the “Priority 1: Critical” category of pathogens. With the increasing prevalence of antibiotic resistance and limited development of new classes of antibiotics, alternative antimicrobial therapies are needed, with lytic bacteriophage (phage) specifically targeted against each of the high priority bacterial infections as a potential approach currently in development toward regulatory approval for clinical use. Balb/c mice were prophylactically administered PBS or phage selected against *A. baumannii* strain AB5075. After 3 weeks, mice were anesthetized, wounded (dorsal), and challenged topically with AB5075. Following infection, mice were subsequently treated with PBS or phage for three consecutive days, and evaluated for 3 weeks to assess the safety and efficacy of the phage treatment relative to the control. We assessed mortality, bacterial burden, time to wound closure, systemic and local cytokine profiles, alterations in host cellular immunity, and finally presence of neutralizing antibodies to the phage mixture. In our study, we found that prophylactic phage administration led to a significant reduction in monocyte-related cytokines in serum compared to mice given PBS. However, we detected no significant changes to circulating blood populations or immune cell populations of secondary lymphoid organs compared to PBS-treated mice. Following prophylactic phage administration, we detected a marked increase in total immunoglobulins in serum, particularly IgG2a and IgG2b. Furthermore, we determined that these antibodies were able to specifically target phage and effectively neutralize their ability to lyse their respective target. In regards to their therapeutic efficacy, administration of phage treatment effectively decreased wound size of mice infected with AB5075 without adverse effects. In conclusion, our data demonstrate that phage can serve as a safe and effective novel therapeutic agent against *A. baumannii* without adverse reactions to the host and pre-exposure to phage does not seem to adversely affect therapeutic efficacy. This study is an important proof of concept to support the efforts to develop phage as a novel therapeutic product for treatment of complex bacterial wound infections.

## Introduction

The Infectious Diseases Society of America (IDSA) has categorized antimicrobial resistance as “one of the greatest threats to human health worldwide” (Hospenthal et al., 2011). The augmented frequency of multi-drug resistant (MDR) bacterial pathogens has been closely associated with the clinical use of broad spectrum antibiotics, thus ensuing an amplified mutation rate in attempts to maintain bacterial survival (Ena et al., 1993). In addition, there is a tremendous financial burden associated with these infections, which has been estimated to be between $21 billion and $34 billion in the United States alone (Spellberg et al., 2013). The primary culprits have been identified as the ESKAPE pathogens (*Enterococcus faecium*, *Staphylococcus aureus*, *Klebsiella pneumoniae*, *Acinetobacter baumannii*, *Pseudomonas aeruginosa*, and *Enterobacter* spp.), and can cause severe local and systemic infections (Murray et al., 2009; Hospenthal et al., 2011). *A. baumannii*, for example, is a Gram-negative, encapsulated opportunistic pathogen that can cause various types of infections, such as pneumonia, bloodstream infections, meningitis, urinary tract infections, wound, and surgical site infections (Safdar and Maki, 2002; Maragakis and Perl, 2008). According to the CDC, it is estimated that 63% of *Acinetobacter* strains are found to be MDR *A. baumannii*^1^ (Center for Disease Control and Pravention, 2013).

A majority of combat-related injuries are associated with significant tissue destruction, and therefore are at high risk for infectious complications, which have occurred in up to 35% of service members who suffered from combat-related injuries during Operations Iraqi- and Enduring Freedom (Murray et al., 2009; Hospenthal et al., 2011). A majority of these infections involved skin, soft tissue, and wounds, and the prevalence of infection in severe injuries, such as open tibial fractures, was as high as 77% (Johnson et al., 2007). Because of the severity of their injuries, patients with combat-trauma are also at a high risk for acquiring nosocomial infections from MDR bacteria due to prolonged hospitalization, inter-institutional transfer, exposure to invasive devices (e.g., central venous catheters, etc.), and exposure to antibiotics (Safdar and Maki, 2002). Pathogens of the ESKAPE (*E. faecium*, *S. aureus*, *Klebsiella* spp., *A. baumannii*, *P. aeruginosa*, and *Enterobacter* spp.) classification were identified as the most frequent isolates associated with combat-trauma wound infections (Keen et al., 2010). The limited number of treatment options against MDR infections is exacerbated by the continuing shortage of new antibiotics in the development pipeline (Spellberg et al., 2013), thus alternative therapeutic are urgently needed to treat complex infections in both military and civilian populations.

Lytic bacteriophage (phage) are viruses that specifically infect and lyse bacteria. Phages have long been considered for use in therapy based on advantages including the high specificity of phage for the host bacteria, the exponential accumulation of phage when bacteria are present, and rapid removal of phage after bacterial clearance. Currently phage therapy is used to prevent and treat bacterial infections in the countries of Georgia and Poland, but the clinical studies with phage in these countries lack quantitative information and adequate controls to robustly address the safety and efficacy of phage therapy (Boucher et al., 2009). In the current study, we investigated the efficacy of phage as a prophylactic and therapeutic regimen, and the impact that pre-immunization to phage has on subsequent efficacy.

## Materials and Methods

### Mice

Female 6-week-old BALB/c mice were purchased from Charles River. Mice were quarantined for 2 weeks prior to the start of the study. Mice were single housed at the start of the study for 1 week, before being group housed.

### Ethics Statement

Research was performed in compliance with the Animal Welfare Act and adheres to principles stated in the *Guide for the Care and Use of Laboratory Animals* (Council, 2011). The study protocol was reviewed and approved by the Institutional Animal Care and Use Committee at the Walter Reed Army Institute of Research and Naval Medical Research Center in compliance with all applicable Federal regulations governing the protection of animals in research.

### Reagents and Monoclonal Antibodies

The reagents were purchased as follows: red blood cell lysis buffer from Sigma-Aldrich (St. Louis, MO, United States); Percoll, phosphate-buffered saline (PBS) and fetal bovine serum (FBS) from Fisher Scientific; fixation solution from Biolegend.

The following anti-mouse monoclonal antibodies were purchased from BioLegend (San Diego, CA, United States): CD3 APC-Cy7 (17A2), CD4 Brilliant Violet 605 (GK1.5), CD8a PE-Cy7 (53-6.7), CD19 FITC (6D5), TCRγδ PE (GL3), and F4/80 Brilliant Violet 510 (BM8). The following anti-mouse monoclonal antibodies were purchased from BD Biosciences (San Diego, CA, United States): CD11c APC-R700 (N418). The following anti-mouse monoclonal antibodies were purchased from Bio-Rad (Hercules, CA, United States): Ly6B.2 Alexa Fluor 647 (7/4). The following anti-mouse monoclonal antibodies were purchased from Thermo Fisher Scientific: Live/Dead Fix Blue (L23105).

### Bacterial Strains and Phage Mixture

In order to visualize wound bacterial burden during animal studies, in the current study, the bioluminescent strain AB5075 *att*Tn*7*:*luxCDABE* (AB5075:lux) (see Supplementary Material for antibiotic susceptibility profile), previously described by Regeimbal et al. (2016), was used to inoculate and infect naïve Balb/c. AB5075:lux, which was used to determine wound bacterial burden via luminescent signal, was maintained on tryptic soy broth (TSB; Becton, Dickinson and Company) or Lennox LB broth (Becton, Dickinson and Company) and stored in 20–40% glycerol at −80°C. The five member phage mixture (AbArmy ϕ1, AbNavy ϕ1, AbNavy ϕ2, AbNavy ϕ3, and AbNavy ϕ4) that was designed to be lytic against AB5075:lux was added at equal volumes to generate a stock concentration of 1 × 10^9^ PFU/mL and was described previously by Regeimbal et al. (2016).

### Immunophenotyping

Various tissues [spleen, lymph nodes (LN), and liver] were collected at various time points from naïve mice following administration of either PBS (control) or AB5075 phage mixture to determine changes to the frequency of different immune cell populations. Tissues were homogenized into a single cell suspension. Mononuclear cells from liver homogenates were enriched using a 33% Percoll gradient. Tissue homogenates were subjected to red blood cell lysis and total cell count enumerated using a BioRad automated cell counter. For analysis, 2 × 10^6^ cells were stained with Live/Dead Fix Blue according to the manufacturer’s protocol, followed by immunostaining with anti-mouse monoclonal antibodies target against extracellular markers to various immune cell populations for 20 min at 4°C in the presence of 1× PBS supplemented with 2% FBS. Cells were then washed twice, fixed with 4% paraformaldehyde 20 min at 4°C, washed again, and finally suspended in PBS. All samples were ran on a BD LSR Fortessa (BD Biosciences, San Diego, CA, United States) by collecting ≥10,000 events and analyzed using FlowJo software. Negative gates were set using fluorescence-minus-one controls. The following gating strategy was used to assess different immune cell populations: SSC/FSC panel was gated on cells to exclude debris → singlets were gated → live cells only (based on the live/dead stain). From the live cell gate, CD3 (alone), CD11c, CD19, and F4/80 were then assessed. Subsets of CD3+ cells were further differentiated based on the positive gated population CD3 to determine CD3+/CD4+, CD3+/CD8+, and CD3+/γδTCR+ populations.

### Phage Neutralization Assay

Serum collected from at various time points from naïve mice following administration of either PBS (control) or AB5075 phage mixture were stored in a −80°C freezer until the assay was performed. Serum was serially diluted in 96-well microtiter plates. AB5075 phage mixture was diluted to a titer of 2 × 10^6^ PFU/mL in PBS. Phage (4 × 10^4^ total PFU) was incubated at 37°C with respective serum at a 1:10 ratio. Samples of the incubated phage-serum mixture were taken at various time intervals and further diluted 1:100 in PBS to stop the antibody reaction. The diluted PBS-phage-serum mixture was spot plated on a lawn of *A. baumannii* and assessed for plaque formation. The neutralization rate (*K*-value) was determined by the following equation to indicate the rate of 90% phage inactivation as described in Lusiak-Szelachowska et al. (2014): *K* = (2.3 *D*/*t*) × log (*p*_0_/*p*).

### Cytokine/Chemokine Analysis

Serum cytokines/chemokines levels were assessed at various time points from naïve mice following administration of either PBS (control) or AB5075 phage mixture. A custom Millipore 25-plex Mouse Cytokine/Chemokine Magnetic Bead Panel kit (MCYTOMAG-70K-PMX, Burlington, MA, United States) was purchased and used according to the manufacturer’s instructions to analyze the following: G-CSF, IFNγ, IL-1α, IL-1β, IL-2, IL-4, IL-5, IL-6, IL-7, IL-9, IL-10, IL-12p40, IL-12p70, IL-13, IL-15, IL-17, IP-10, KC, MCP-1 MIP-1a, MIP-1b, MIP-2, RANTES, TNFα, and GM-CSF.

### Total Immunoglobulin Measurements

Total immunoglobulins for serum were assessed at various time points from naïve mice following administration of either PBS (control) or AB5075 phage mixture. A custom Millipore Mouse Immunoglobulin Isotyping Magnetic Bead Panel kit (MGAMMAG-300K, Burlington, MA, United States) was purchased and used according to the manufacturer’s instructions to analyze the following: IgA, IgG1, IgG2a, IgG2b, IgG3, and IgM.

### Full Thickness Dorsal Wound Mouse Model

**FIGURE 1:**
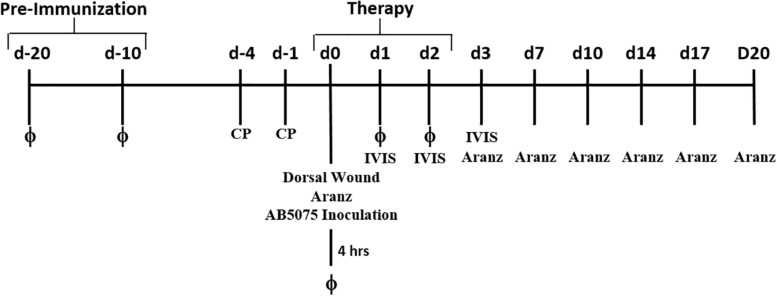
Experimental timeline. Diagram of experimental treatments and model design.

Phage treatment was assessed using a previously described mouse wound model (Thompson et al., 2014) with some modifications. Briefly, 6-week-old female BALB/c mice were randomly separated and administered either 100 μL of PBS or 1 × 10^9^ PFU (1 × 10^8^ total PFU) of phages in PBS intraperitoneally (i.p.) at Days 20 and 10 (Figure 1). Mice were immunosuppressed via cyclophosphamide injection on Days 4 and 1. On Day 0, their backs were shaved, and a full-thickness wound was created on the dorsal side of each mouse using a sterile 6 mm biopsy punch (∼0.3 cm^2^). Each wound was inoculated with ∼5 × 10^6^ total CFU of AB5075:lux, and a Tegaderm bandage was placed over the wound. Mice were housed singly from day 0 (inoculation) to day 6. Mice were then further separated into four group based on their pre-treatment/post-treatment regimen: (1) PBS–PBS, (2) PBS–Phage, (3) Phage–PBS, and (4) Phage–Phage. For phage treatments, mice received both 100 μL of 1 × 10^9^ PFU (1 × 10^8^ total PFU) of phages in PBS i.p. and 50 mL 1 × 10^9^ PFU (5 × 10^7^ total PFU) of phages in PBS delivered topically under the Tegaderm dressing, on top of the wound. Treatments were administered at 4, 24, and 48 h post-infection. On day 6 post-infection, each Tegaderm dressing was removed and the wounds were left exposed to air for the remainder of the experiment. An IVIS *in vivo* imaging system (IVIS) (PerkinElmer, Waltham, MA, United States) was used to measure the bioluminescent signal of AB5075:lux as a means to visualize and perform relative quantification of bacterial burdens in the wound beds of anesthetized mice over the course of the experiment. Living Image Software version 4.2 (PerkinElmer, Waltham, MA, United States) was used to analyze and quantify *in vivo* bioluminescence as defined with the “Auto ROI” function with a threshold of 10%. The Aranz Silhouette wound measurement device was used to image and measure the physical wound size of mice at various time points throughout the study (Aranz Medical Ltd., Christchurch, New Zealand).

### Detection of Phage Titers in Organs

Naïve 6-week-old female BALB/c mice were randomly separated and administered either 100 μL of PBS or 1 × 10^9^ PFU (1 × 10^8^ total PFU) of phages in PBS i.p. at Days 20 and 10 (Figure 1). Mice were euthanized at Days 19, 15, and 5, and various organs were excised for the presence of phage. Tissues were homogenized in 5 mL PBS, dilutions of 10^−1^−10^−8^ were generated, and the various dilutions were spot plated on a lawn of *A. baumannii* AB5075:lux. Samples were allowed to incubate at 37°C overnight and phage plaques were enumerated the next day.

### Statistical Analysis

The data shown in this paper is pooled from at least three independent experiments. The mean ± standard error of the mean (SEM) is shown for experiments that are applicable. The statistical differences within experiments were calculated using an analysis of variance (ANOVA). A *P-*value < 0.05 was considered to be statistically significant.

## Results

### Distribution of Phage Upon Administration

**FIGURE 2:**
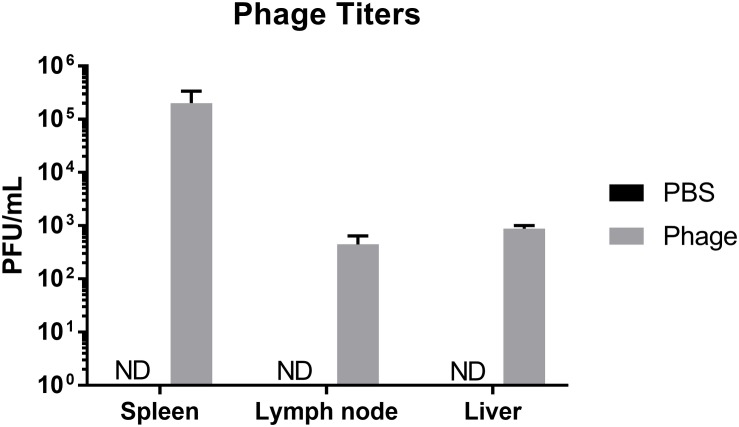
Distribution of phage upon administration. Naïve Balb/c mice were assessed for phage titers 24 h after PBS or phage mixture administration. *N* = 7 per group (ND = not detected).

Phage are viruses that have evolved to interact and target a specific bacterial host. Considering the diverse composition of phage, in addition to their ability lyse select bacterial strains and subsequently release components of lysed bacterial cells into the environment, phage may either directly or indirectly influence the mammalian host immune system. In order to better understand the immunogenicity and limitations of phage therapy, we wanted to first determine where phage migrate upon systemic administration. Therefore, we administered either sterile PBS or 10^8^ total PFU of a five member AB5075 phage mixture i.p. to naïve Balb/c mice. After 24 h, we collected peripheral tissues, such as spleen, liver, and lymph nodes (LN) pooled from throughout the body, and examined them for the presence of phage. We found in mice administered phage, the spleen possessed the highest titer of phage (2.02 × 10^5^ ± 1.34 × 10^5^ PFU/mL) followed by liver (8.71 × 10^2^ ± 1.29 × 10^2^ PFU/mL) and LN (4.43 × 10^5^ ± 1.97 × 10^2^ PFU/mL), whereas control animals had undetectable titers in all organs (Figure 2).

Phage Mixture Administration Decreases Monocyte-Related Cytokines/Chemokines in Serum.

**FIGURE 3:**
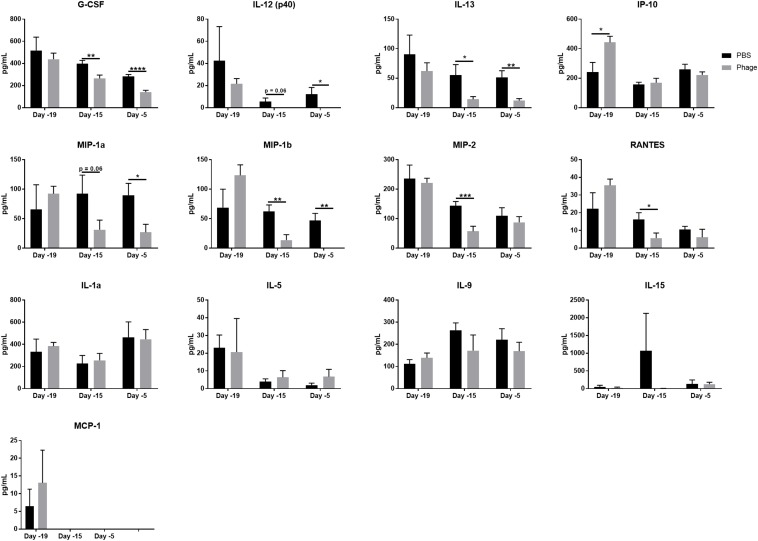
Phage mixture administration decreases monocyte-related cytokines/chemokines in serum. Serum collected from naïve mice given PBS (control) or phage mixture were analyzed for cytokines/chemokines using a 25-plex panel. *N* = 10 per group for each time point; *p < 0.05, **p < 0.01, ***p < 0.001, and ****p < 0.0001.

Next, we sought to determine whether the AB5075 phage mixture impelled cytokine and chemokine production following systemic administration. Thus, we administered either 100 μL of sterile PBS or 1 × 10^9^ PFU of the AB5075 phage mixture i.p. to naïve Balb/c mice at Days 20 and 10, and collected serum at various time points (Days 19, 15, and 5) (Figure 3). Of the 25 cytokines/chemokines in our panel, only 8 illustrated a difference between PBS- and phage-treated mice (Figure 3). These circulating factors included G-CSF, IL-12 (p40), IL-13, IP-10/CXCL10, MIP1α, MIP1β, MIP2, and RANTES, which have been shown to be associated with the maturation and function of monocytes. Interestingly, the AB5075 phage mixture was observed to significantly downregulate these eight cytokine/chemokine levels in serum within 5 days of a single administration to naïve mice compared to PBS control. Moreover, some of their levels were further decreased following a second administration. We found no significant differences in IL-1a, IL-5, IL-9, IL-15, and MCP-1 between PBS and phage administered naïve mice (Figure 3). The remaining 12 cytokines/chemokines in the panel, which included prominent pro-inflammatory markers IFNγ and TNFα as well as anti-inflammatory markers IL-4 and IL-10, were measured as below detectable levels.

### Administration of Phage Mixture Does Not Modulate Immune Cell Populations

**FIGURE 4:**
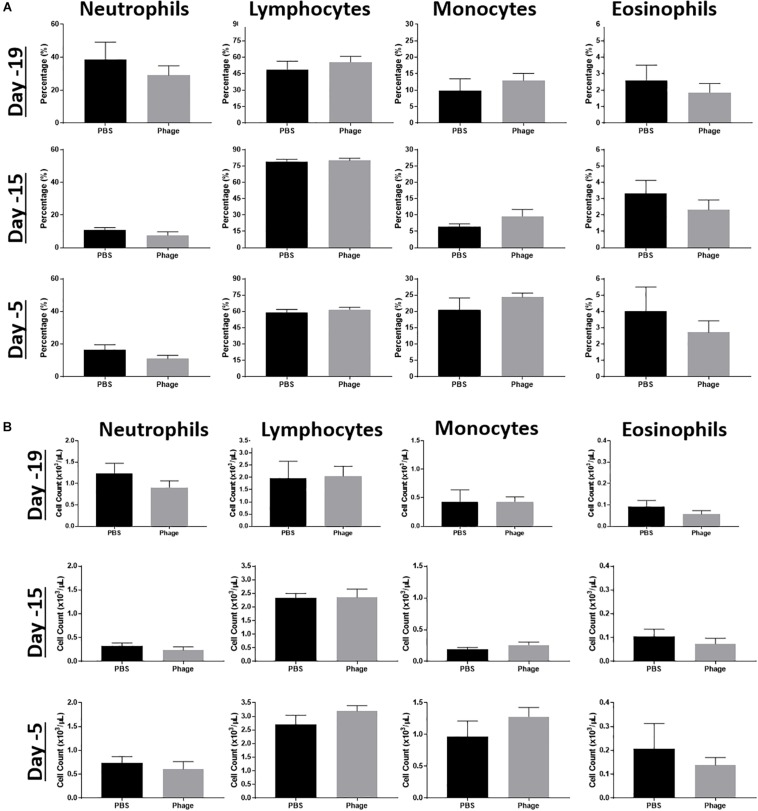
Effects of phage mixture on peripheral immune cell populations. Complete blood counts, **(A)** percentages and **(B)** cell count were measured from whole blood at various time points following PBS (control) or phage mixture administration in naïve mice. *N* = 6 per group per time point.

Due to the altered cytokine/chemokine profile following systemic administration of the AB5075 phage mixture, we wanted to establish its potential effects on other facets of the immune system. Thus, we collected whole blood at various time points from naïve mice administered PBS or AB5075 phage mixture at Days 20 and 10. Subsequently, we examined their complete blood count (CBC) profile, focusing on the frequency and total cell number of immune cell populations, such as neutrophils, monocytes, lymphocytes, and eosinophils (Figure 4). As a result, we observed no statistical differences or trends between groups at any of the time points collected.

**FIGURE 5:**
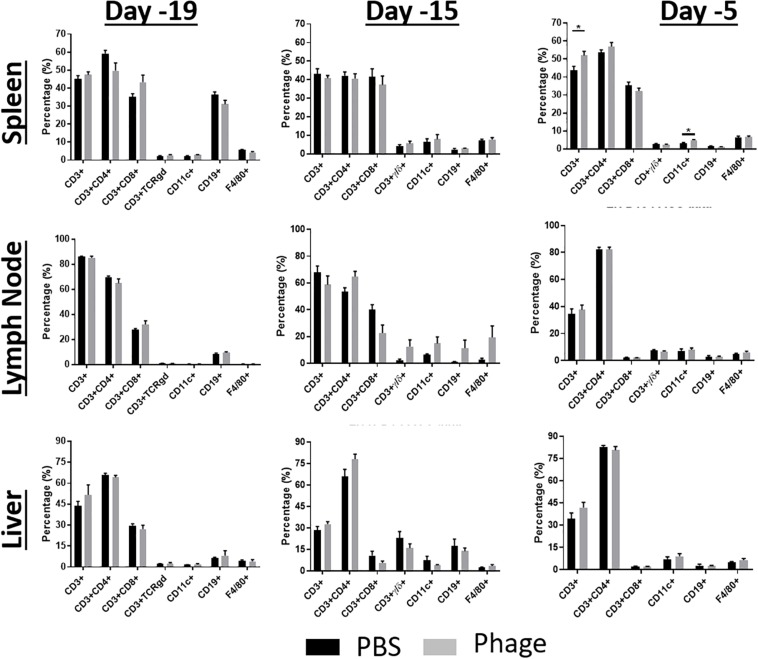
Administration of phage mixture does not modulate immune cell populations. Immune cell populations were assessed at various time points from naïve mice following PBS (control) or phage mixture administration (i.p.). *N* = 6 per group per time point; *p < 0.05.

After seeing no impact on systemic immune cell populations, we took a closer look at cell populations that reside in secondary lymphoid tissues, such as spleen, LN, and liver (Figure 5). We examined these tissues for any gross changes that may occur in the prominent immune bionetwork using flow cytometry. Similar to what we observed in the CBC profiles, AB5075 phage mixture appeared to elicit no significant impact on the frequency of T-cells (CD3^+^: CD4^+^, CD8^+^, TCRγδ^+^), B-cells (CD19^+^), dendritic cells (CD11c^+^), or macrophages (F4/80^+^) in any of the tissues.

### Phage Mixture Administration Promotes IgG2a and IgG2b Antibody Generation

**FIGURE 6:**
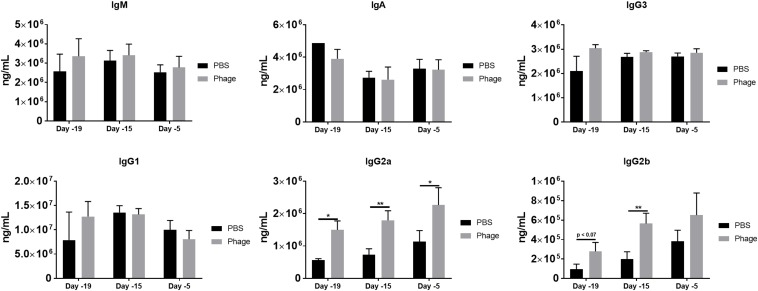
Phage mixture administration promotes IgG2a and IgG2b generation. Serum collected from naïve mice given PBS or phage mixture were analyzed for total immunoglobulins. *N* = 10 per group for each time point; *p < 0.05, **p < 0.01.

Unlike cytokines, which can be produced and secreted by any cell type, antibodies, or immunoglobulins can only be derived from plasma B-cells. In the current study, we investigated immunoglobulin levels in circulation following single or multiple administrations of PBS or AB5075 phage mixture (Figure 6). We found serum IgM levels to sustain around ∼3 × 10^6^ ng/mL at the various time points for either PBS or AB5075 phage mixture treated mice. Similarly, AB5075 phage mixture administration did not alter the immunoglobulin titers IgA, IgG1, or IgG3 compared to PBS treated mice on a day-to-day basis (Figure 6). Surprisingly, mice given AB5075 phage mixture revealed a prompt induction of antibody titer for both Ig2a and Ig2b within 24 h of administration in comparison to PBS mice. Moreover, Ig2a and Ig2b serum levels continued to be enhanced 5 days after the first administration of AB5075 phage mixture given on Day 20 and continued to be augmented following the second administration on Day 10.

### Detection of Serum Neutralizing Antibodies Against *A. baumannii* Phage Mixture

**FIGURE 7:**
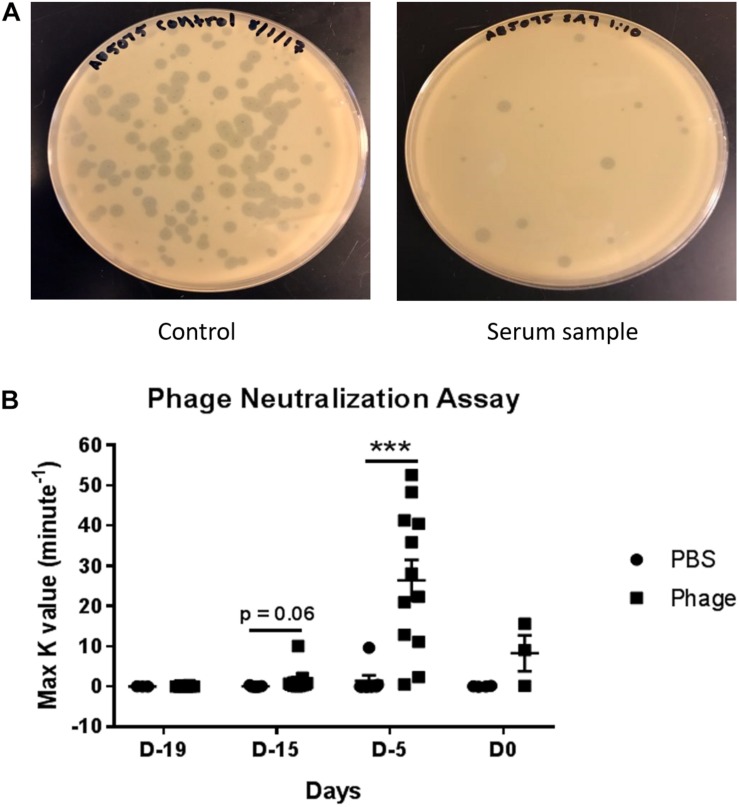
Detection of serum neutralizing antibodies against *A. baumannii* phage mixture. Dilutions were made from serum collected from naïve mice given PBS (control) or phage mixture and incubated with a known titer of phage mixture (4 × 10^4^ total PFU). **(A)** The mixtures were then added to an inoculum of AB5075 and plated in warm agar. **(B)** After 24 h incubation at 37°C, rate of phage neutralization was measured. *N* = 7 for PBS, *N* = 13 for Phage per time point; ***p < 0.001.

Upon detecting significant amounts of immunoglobulin titers in serum following AB5075 phage mixture administration, we set out to determine whether those antibodies specifically targeted our phage mixture as well as potentially impeded the mixture’s therapeutic functions and efficacy (Figure 7). In testing for the presence of neutralizing antibodies, we diluted a set of previously collected serum samples from PBS or AB5075 phage treated mice. The diluted serum was then incubated with a known titer of AB5075 phage mixture (4 × 10^4^ total PFU) before being added to a lawn of AB5075. After overnight incubation, the agar plates were assessed for plaque formation, which would indicate the ability of the phage mixture to bind and lyse that targeted bacteria. Accordingly, a *K*-value was calculated to signify the rate at which 90% phage were neutralized and unable to lyse their intended target compared to unhindered phage titer control. Interestingly, despite the fact that we were able to detect high titers of immunoglobulins as early as Day 19, we were only able to detect the initial trend of neutralizing antibodies at Day 15 as illustrated by the *K*-value of 1.511 ± 0.869 max *K*/min observed from AB5075 phage treated mice compared to 0.058 ± 0.061 max *K*/min of PBS-treated mice. More importantly, the occurrence of neutralizing antibodies was found to peak markedly at Day 5, where the *K*-value was roughly 18-fold greater in AB5075 phage treated mice compared to those given PBS (AB5075 phage: 26.36 ± 5.063 max *K*/min vs. PBS: 1.408 ± 1.372 max *K*/min). Surprisingly, this notable increase hastily appeared to subside to almost baseline by Day 0 (AB5075 phage: 8.262 ± 4.469 max *K*/min vs. PBS: 0.047 ± 0.046 max *K*/min).

### Effects of Phage Mixture on Wound Closure and Bacterial Burden Following *A. baumannii* Infection

The presence of a bacterial infection compounds the stresses and burdens placed on the host’s immune system. Moreover, in the case of an open wound, it can prolong the wound healing process and exacerbate inflammation and tissue damage. As an alternative approach to standard of care antibiotics, our institute has previous shown that phage therapy may serve as a selective and effective tool against bacterial pathogens, even those that have become resistant to current treatment regimens (Regeimbal et al., 2016). The potential caveat in phage therapy lies in the host’s immune system generating a response against the phage following multiple treatment regimens and clearing them before the treatment has a chance of clearing the infection. This is important to note given the fact that in the current study, we not only observed a significant increase in the presence of immunoglobulins, particularly IgG2a and IgG2b, but we also found that these serum antibodies were able to neutralize the lytic function of phage in our *in vitro* assays. Therefore, we wanted to evaluate the therapeutic effects of our phage mixture during an *A. baumannii* wound infection following single and multi-dose regimens.

**FIGURE 8:**
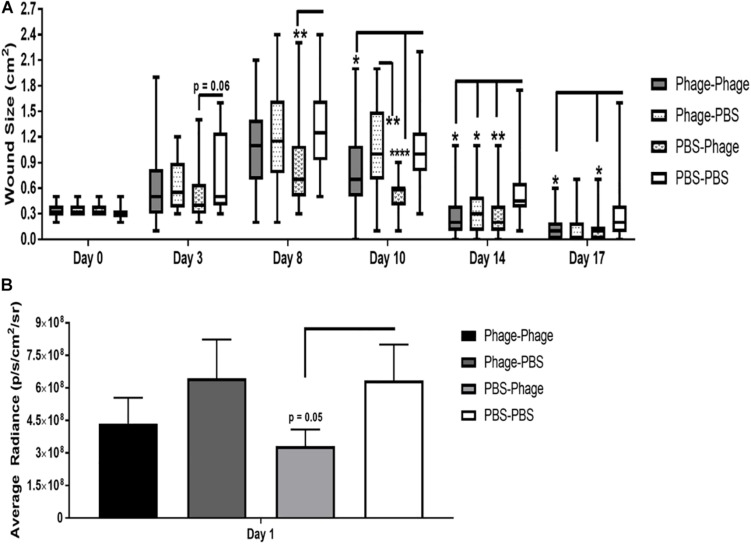
Effects of phage mixture on wound closure and bacterial burden following *A. baumannii* infection. Balb/c mice pre-immunized with PBS or phage mixture were immunosuppressed with cyclophosphamide before wounding (dorsal full thickness injury) and challenged topically with AB5075-lux. **(A)** Wound size measure by Aranz system. **(B)** Bacterial burden measured by IVIS. *N* = 27 per group per time point; *p < 0.05, **p < 0.01, ****p < 0.0001.

In our mouse dorsal wound infection model, we randomly separated mice into four groups where PBS or AB5075 phage mixture were given as prophylactic (in parentheses) or post-infection/treatment regimens in various combinations: PBS–PBS, Phage–PBS, PBS–Phage, and Phage–Phage. Prophylactic regimens were administered i.p. to naïve mice at Days 20 and 10 with either 100 μL of PBS or AB5075 phage mixture (1 × 10^8^ total PFU). Mice were moderately immunosuppressed using cyclophosphamide standardized in our model prior to introducing a 6-mm dorsal wound and subsequent topical infection with *A. baumannii* strain AB5075: *lux*. Post-infection treatment regimens were administered both i.p. and topically at 4 h after infection on Days 0, 1, and 2. We found that PBS–PBS-treated mice subsequently infected with AB5075: *lux* had a high bacterial bioburden 24 h after infection as measured by capturing the bacterial luminescence signal using an IVIS. Bacterial burden did not appear to be altered by prophylactic administration of AB5075 phage mixture, designated as Phage-PBS (Figure 8B). The treatment regimen of AB5075 phage mixture [PBS-Phage], on the other hand, was able to promptly reduce bacterial bioburden compared to PBS–PBS mice (3.32 × 10^8^ ± 8 × 10^7^ vs. 6.35 × 10^8^ ± 2 × 10^8^ p/s/cm^2^/sr) (Figure 8A). Meanwhile, the additive measures of including both a pre- and post-infection treatment regimen, Phage–Phage, provided an intermediary effect between PBS–PBS and PBS–Phage mice. In regards to wound size following infection with AB5075: *lux*, we observed that wounds of PBS–PBS mice rapidly increased from 0.3 ± 0.01 cm^2^ on Day 0 to 1.27 ± 0.09 cm^2^ by Day 8. Furthermore, wounds of PBS–PBS mice only returned to size of the original injury (0.3 ± 0.01 cm^2^) around Day 17, but never closed by the end of the study (Figure 8B). In comparison, Phage–PBS mice exhibited a similar healing profile and wound sizes as PBS–PBS mice throughout the study, suggesting the prophylactic regimen of AB5075 phage mixture was not effective in mitigating the bioburden and collateral damage of the infectious pathogen. PBS–Phage mice, however, showed reduced wound size throughout the study, especially at the apex on Day 8 in which overall wound sizes were limited to 0.832 ± 0.0.09 cm^2^. Additionally, wounds of PBS–Phage not only returned to baseline by Day 14, which was a full 3 days before PBS–PBS mice, but were fully closed by Day 17. When we examined if there was a change in efficacy following multiple administrations of the same phage mixture, we found that Phage–Phage mice had condensed wound sizes, peaking on Day 8 at 0.9 ± 0.0.09 cm^2^. Similar to PBS–Phage mice, Phage–Phage mice also presented wounds that returned to original size by Day 14 and complete closure by Day 17.

## Discussion

The prevalence of MDR bacterial pathogens is a growing threat to public health due to a variety of factors, such as immune competence, educational awareness and intervention, and overuse of antibiotics in food products and patients. Thus, there is an urgent need for new therapeutics against the MDR pathogens, including *A. baumannii*, that either replace or work in tandem with current standard of care. Recently, phages have reemerged as potential antibacterial candidates due to their narrow spectrum and targeted actions. In the current study, we demonstrate that phages can serve as a safe and effective novel therapeutic agent against *A. baumannii* when given as a single or sequential regimen without adverse reactions. Moreover, this study helps provide a deeper understanding of the interactions between host immune, bacteria, and phage to better predict bacterial pathogenesis and further develop phages as a novel and effective therapeutic product for treatment of complex infections in military beneficiaries.

The microbiota of healthy humans comprises of a variety of bacterial populations as well as a large number of bacterial viruses, such as phages (Reyes et al., 2012). Ironically, one of the primary benefits of using phage as an antibacterial therapeutic is that they are extremely narrow spectrum, even to the subspecies level, which minimizes the potential damage to the host microbiome. Phages have been found to colonize sites all over the body, including the skin (Foulongne et al., 2012; Oh et al., 2016), oral cavity (Pride et al., 2012), lungs (Dickson and Huffnagle, 2015), gut (Reyes et al., 2010; Minot et al., 2011), urinary tract (Santiago-Rodriguez et al., 2015), and blood (Breitbart and Rohwer, 2005). In the gut, for example, it is estimated that there are 10^9^ viruses per gram of feces (Kim et al., 2011; Reyes et al., 2013), which equates to approximately 90% of the gut virome being phages (Scarpellini et al., 2015). Furthermore, studies have shown that phage can enter and cross different types of epithelial cell layers (e.g., gut, lung, liver, kidney, and brain cells) and for diverse phage types and morphologies (e.g., *Myoviridae*, *Siphoviridae*, and *Podoviridae*) by a non-specific transcytosis mechanism (Nguyen et al., 2017). Thus, it is important to recognize and determine the potential interactions between phage and commensal bacteria, and how their co-inhabitance impact the function of the immune system and influence the spread of pathogens (Duerkop and Hooper, 2013).

In the current study, we detected that a significant amount of phage transmigrated to secondary lymphoid organs, such as the spleen, liver, and LN, within 24 h following intraperitoneal administration in naïve mice. Similarly, other studies have also found a high degree of phage titers in the spleen and liver, primarily attributed to the mononuclear phagocyte system (MPS) (Nungester and Watrous, 1934). The MPS, which helps filter and neutralize foreign objects from circulation (Hodyra-Stefaniak et al., 2015), has also been reported to rapidly remove wild-type phage λ from the circulatory system following administration in humans (Geier et al., 1973). As a part of the phagocytosis process, phages demonstrated the ability to enhance phagocytosis either by opsonization of bacterial cells or release of contributing factors during the degradation (Kaur et al., 2014).

In a number of studies, phages have demonstrated the ability to induce cytokine and chemokine production. However, it is sometimes difficult to determine the extent of phage influence due to purified phage vs. lystates, bacterial contaminants (e.g., LPS, cytosolic proteins, or membrane particles), and types of phage being investigated (Weber-Dabrowska et al., 2000; Kaur et al., 2014). In our study, we found that our five-member *A. baumannii* phage mixture did not appear to alter any of the mainstream pro-inflammatory and anti-inflammatory cytokines, such as IFNg, TNFα, IL-6, and IL-10. Interestingly, our phage mixture was able to significantly decrease 10 serum cytokines/chemokines in naïve mice that are associated with monocyte maintenance and function. Current literature supports the notion of phages’ ability to modulate cytokine responses upon treatment. Park et al. (2014), for example, demonstrated that mice fed phage T7 daily for 10 days only led to subtle increases in inflammatory cytokine production and no significant histopathological changes. Similarly, mice i.p. administered highly purified preparations of either whole phage T4 particles, or four phage T4 capsid proteins (i.e., gp23^∗^, gp24^∗^, Hoc, and Soc) expressed no inflammatory cytokines (Miernikiewicz et al., 2013). In a 2000 study, 51 patients with long-term suppurative infections of various tissues and organs caused by drug-resistant strains of bacteria were treated with phage (Miernikiewicz et al., 2013). As a result, the patients treated with phage exhibited a significant reduction in TNF-a and IL-6, which may have also been attributed to the decreased number of pathogenic bacteria in the body following therapeutic application of the phage. In another study, five highly purified phages targeting two different pathogens, *P. aeruginosa* and *S. aureus*, were shown to augment suppressor of cytokine signaling 3 (SOSC3), IL-1 receptor antagonist (IL1RN), and IL-6 levels in PBMCs derived from healthy human donors (Van Belleghem et al., 2017). Meanwhile, evidence suggest that *S. aureus* phage, vB_SauM_JS25, is able to suppress LPS-induced inflammation (Zhang et al., 2018).

Recently, it has been suggested that commensal phages may have the potential to stimulate low-level immune responses without causing any overt symptoms as a means to continuously prime innate immune responses (Farrar and Schreiber, 1993; Duerkop and Hooper, 2013). In our study, we observed no significant changes in immune cell populations in circulation or secondary lymphoid organs with our phage mixture. However, Tiwari et al. (2011) have demonstrated the importance of neutrophil-phage cooperation in the resolution of *P. aeruginosa* infections, where the presence of neutrophils was imperative to remove resistant bacteria that emerged during phage treatment. Similar results were achieved by Roach et al. (2017) and Pincus et al. (2015).

Phages have also displayed the ability to induce humoral immunity. Although we were unable to detect any cellular changes upon phage administration in naïve mice, we did find enhanced immunoglobulin levels in serum. There are five major classes of immunoglobulins (IgA, IgD, IgE, IgG, and IgM), which vary in their chemical structure, number of antigen binding sites, and specificity to select antigen. IgA, for example, is dimer that is generally found in high concentrations in the mucous membranes, such as respiratory passages and gastrointestinal tract, as well as in saliva, bile, and tears. IgD, on the other hand, is often detected in only trace amounts in the blood. IgE is primarily found in the lungs, skin, and mucous membranes. Additionally, this immunoglobulin is associated with allergic reactions and results in the release of histamine. IgM, which is found mainly in the blood and lymph fluid, is a pentamer that is responsible for primary antibody responses. As a result, it is the first antibody to emerge upon detection of a foreign antigen and activates the complement system to fight and eliminate new threats. IgG, which includes the subclasses IgG1, IgG2a, and IgG2b, is the most abundant type of antibody. It is generally found in all body fluids and protects against bacterial and viral infections due to its ability to incorporate opsonization and the complement system.

Under “normal” circumstances, naive B cells only express cell-surface IgM and IgD with identical antigen binding regions. Upon introduction to foreign antigen and generation of cytokines, these cells become activated and undergo a process known as class switching. This process induces the expression of IgG, IgA, and IgE, as well as inherent changes in antibody structure, antigen specificity, and overall functionally that allow it to work more effectively and efficiently at clearing present and future infections (Al-Lazikani et al., 1997). In the current study, we found IgG2a and IgG2b titers were significantly increased over time following phage administration, despite no changes in IgM compared to mice given PBS. Subsequently, we were able to determine that the antibodies generated were capable of neutralizing the lytic activity of our phage mixture. However, data suggest that either the presence of the antibodies or neutralizing activity may be transient, given the fact we observed a substantial decrease in neutralizing activity following Day 5. In alignment with our findings, a number of other studies have also detected the presence of phage-neutralizing antibodies against naturally occurring phages (e.g., not therapeutically administered) in the sera of different species (e.g., mice, horse, or human) (Dabrowska et al., 2014). In 2014, Dabrowska et al. (2014) evaluated 50 healthy volunteers who had never been subjected to phage therapy nor involved in phage work for the presence of naturally occurring phage-antibodies against phage T4. As a result, 81% displayed the presence of anti-phage antibodies, which exhibited specificity to phage proteins gp23, gp24, Hoc, and Soc. Interestingly, in a separate study, evidence suggests that phage T4 Hoc protein and gp12 were potent inducers of IgG and IgA antibody production in the blood and gut, respectively, whereas gp23, gp24, and Soc were found to induce low responses (Majerczyk et al., 2010). In a study involving patients that were administered orally or locally the MS-1 phage mixture, which comprises of three lytic *S. aureus* phages, the majority of the patients possessed no detectable presence of neutralizing antibodies (Zaczek et al., 2016). Moreover, the few patients who presented with elevated IgM or IgG levels still had positive clinical outcomes following phage therapy, suggesting these serum neutralizing antibodies did not impede the efficacy of the delivered phage.

In the current study, we demonstrate that upon systemic administration, phage have the propensity to permeate throughout the host’s body, but is quickly sequestered into secondary lymphoid tissues. Based on the abilities of phages to act both as a direct antimicrobials as well as immunomodulatory agents to promote bacterial clearance, they serve as a unique tool capable of complementing and potentially sparing the use of standard of care antibiotics. Although phage are generally characterized as being safe and non-toxic, individual phage and phage mixtures still have the capability of eliciting unique signatures in regards to host responses and the immune system. For example, we observed a rise of select serum cytokines/chemokines and immunoglobulins, as well as induction of neutralizing antibodies upon administration of our AB5075 phage mixture. Recent computational models have been used to depict and elucidate the relationship and interactions between host immunity, phage, and bacteria (Levin and Bull, 2004; Roach et al., 2017). Thus, in a clinical setting, these modulatory effects may influence not just pathogen susceptibility and defenses, but also may dictate the timing and overall efficacy of phage therapy. Collectively, the kinetics of phage propagation vs. bacterial killing can be simplified to five basic parameters: the growth rate and density of the bacteria, the infectivity of the phage, the latency period vs. rate of degradation/removal of phage, the burst size, and the multiplicity of infection of the phage (Levin and Bull, 2004). Understanding these parameters and kinetics will be important to optimize the efficacy of phage therapy in various clinical situations, with patients of different immunocompetency, simultaneous use of different classes of antibiotics of which for some phage may be complementary and others not, and for different types of infections with different pathogens both in monomicrobial and in polymicrobial infections. Our findings, along with others, are highly suggestive of the potential efficacy of phage therapy, even in the presence of a potentially blunting immune response. Further preclinical and clinical studies are needed to elucidate the mechanisms and limitations of phage therapy in order to fulfill the potential promise of phage as a disruptive therapeutic to counter the ever increasing threat of MDR bacterial infections.

## Data Availability Statement

All datasets generated for this study are included in the article/Supplementary Material.

## Ethics Statement

The animal study was reviewed and approved by the Institutional Animal Care and Use Committee at the Walter Reed Army Institute of Research and Naval Medical Research Center.

## Author Contributions

MR designed and performed the experiments, collected and analyzed the data, and prepared the manuscript. JS, JAR, and ML performed the experiments and collected the data. AJ, BB, JR, and MH designed the experiments. MGS designed the experiments and performed the data analysis. MPS designed and performed the experiments, analyzed the data, and prepared the manuscript.

## Disclaimer

The views expressed in this article are those of the authors and do not necessarily reflect the official policies or positions of the Department of the Navy, the Department of the Army, the Department of Defense, or the U.S. Government. The material has been reviewed by the Naval Medical Research Center, and there is no objection to its presentation and/or publication.

## Conflict of Interest

The authors declare that the research was conducted in the absence of any commercial or financial relationships that could be construed as a potential conflict of interest.

## Footnotes

1 https://www.cdc.gov/drugresistance/pdf/ar-threats-2013-508.pdf

## Supplementary Material

FIGURE S1 Detection of serum neutralizing antibodies against individual phage comprising the *A. baumannii* phage mixture. Dilutions were made from serum collected at Day 5 from naïve mice given PBS (control) or phage mixture and incubated with a known titer of the individual phage that comprised the phage mixture phage mixture (4 × 10^4^ total PFU), respectively. The mixtures were then added to an inoculum of AB5075 and plated in warm agar. After 24 h incubation at 37°C, rate of phage neutralization was measured.

TABLE S1 Antibiotic susceptibility profile of *A. baumannii* AB5075.
