# Supplementary figures and images for: Impact of Frequent Administration of Bacteriophage on Therapeutic Efficacy in an A. baumannii Mouse Wound Infection Model
Source: Front Microbiol. 2020 Mar 17;11:414. doi: 10.3389/fmicb.2020.00414 (PMC7090133; doi:10.3389/fmicb.2020.00414)

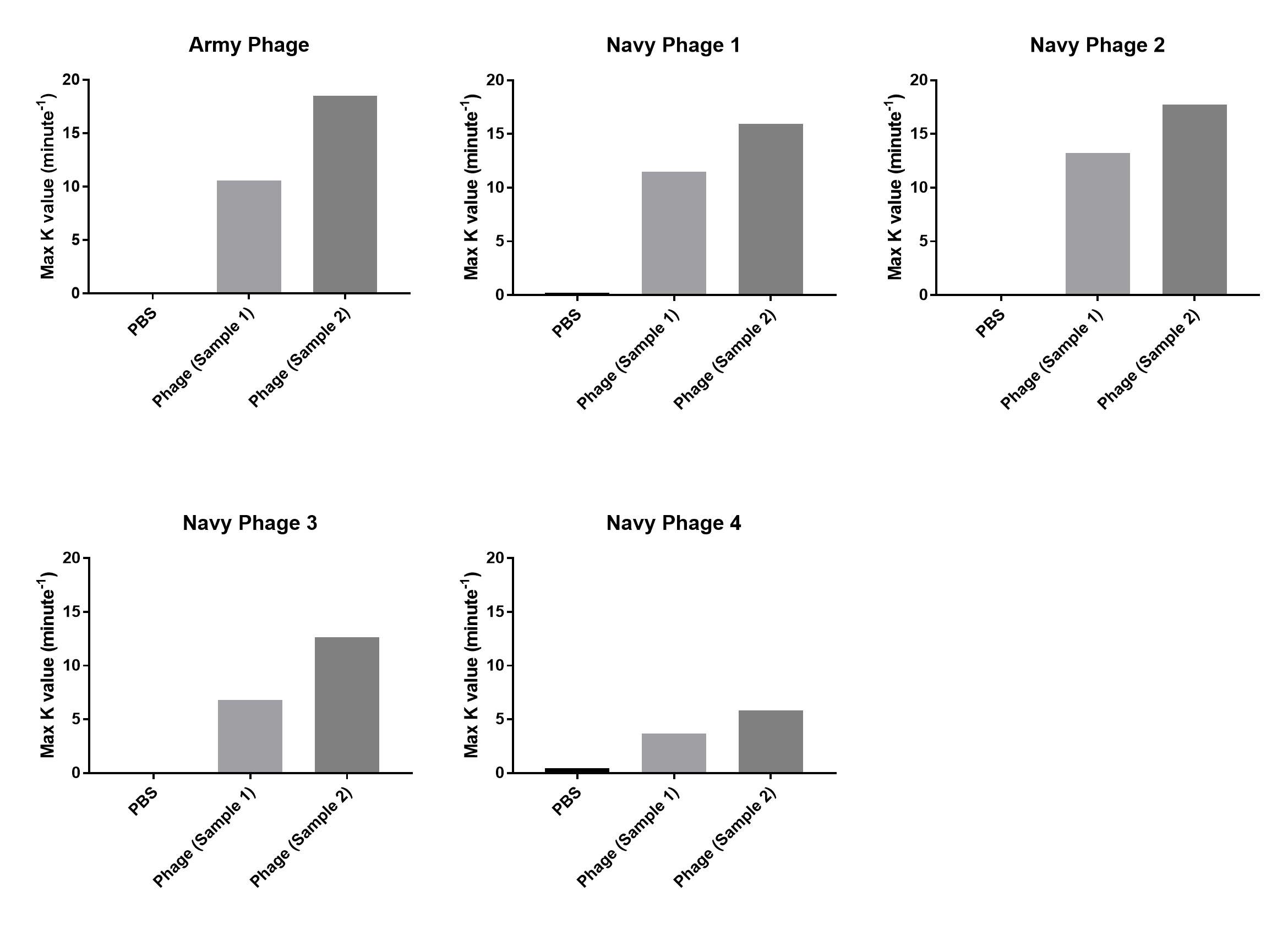

Supplement: FIGURE S1 — Detection of serum neutralizing antibodies against individual phage comprising the A. baumannii phage mixture. Dilutions were made from serum collected at Day 5 from naïve mice given PBS (control) or phage mixture and incubated with a known titer of the individual phage that comprised the phage mixture phage mixture (4 × 10^4^ total PFU), respectively. The mixtures were then added to an inoculum of AB5075 and plated in warm agar. After 24 h incubation at 37°C, rate of phage neutralization was measured. [file Image_1.TIF]
