# Supplementary material for: Impact of Frequent Administration of Bacteriophage on Therapeutic Efficacy in an A. baumannii Mouse Wound Infection Model
Source: Front Microbiol. 2020 Mar 17;11:414. doi: 10.3389/fmicb.2020.00414 (PMC7090133; doi:10.3389/fmicb.2020.00414)
Supplement: TABLE S1 — Antibiotic susceptibility profile of A. baumannii AB5075. [file Table_1.DOCX]

**Phoenix Antibiotic Susceptibility Profile of *A. baumannii* AB5075**

Organism Name: ACINBCX – Acinetobacter baumannii/calcoaceticus complex

| **Drug** | **ACINBCX** | |
| --- | --- | --- |
|  | *MIC/Conc* | *SIR* |
| Amikacin | >32 | R |
| Ampicillin-Sulbactam | >16/8 | R |
| Cefepime | >16 | R |
| Ceftazidime | >16 | R |
| Ceftriaxone | >32 | R |
| Ciprofloxacin | >2 | R |
| Ertapenem | - | R |
| Gentamicin | >8 | R |
| Levofloxacin | >4 | R |
| Meropenem | >8 | R |
| Tetracycline | <=2 | S |
| Tobramycin | >8 | R |
| Trimethoprim-Sulfamethoxazole | >2/38 | R |
